# Dissecting the Antimicrobial Composition of Honey

**DOI:** 10.3390/antibiotics8040251

**Published:** 2019-12-05

**Authors:** Victoria C. Nolan, James Harrison, Jonathan A. G. Cox

**Affiliations:** School of Life and Health Sciences, Aston University, Aston Triangle, Birmingham B4 7ET, UK; 180208508@aston.ac.uk (V.C.N.); j.harrison11@aston.ac.uk (J.H.)

**Keywords:** honey, antimicrobials, methylglyoxal, hydrogen peroxide, bee-defensin 1, wound treatment

## Abstract

Honey is a complex sweet food stuff with well-established antimicrobial and antioxidant properties. It has been used for millennia in a variety of applications, but the most noteworthy include the treatment of surface wounds, burns and inflammation. A variety of substances in honey have been suggested as the key component to its antimicrobial potential; polyphenolic compounds, hydrogen peroxide, methylglyoxal and bee-defensin 1. These components vary greatly across honey samples due to botanical origin, geographical location and secretions from the bee. The use of medical grade honey in the treatment of surface wounds and burns has been seen to improve the healing process, reduce healing time, reduce scarring and prevent microbial contamination. Therefore, if medical grade honeys were to be included in clinical treatment, it would reduce the demand for antibiotic usage. In this review, we outline the constituents of honey and how they affect antibiotic potential in a clinical setting. By identifying the key components, we facilitate the development of an optimally antimicrobial honey by either synthetic or semisynthetic production methods.

## 1. Introduction

Honey has been established as an effective antimicrobial and antioxidant for millennia [1]. Used mainly for the treatment of surface wounds, burns and inflammation, it has since been developed into medical treatments in the form of medical grade honey [2,3]. Despite this, the initial interest into honey as an antimicrobial therapy was drastically diminished upon the discovery and implementation of antibiotics. However, with the alarming rise in the prevalence of antimicrobial-resistant organisms, in particular the increase in multi-drug resistance (MDR), the number of effective antibiotic compounds is shrinking at a greater rate than new drugs are being developed [4,5]. This grave predicament has many researchers looking back to the pre-antibiotic era for solutions, sparking more recent interest into the mechanisms of action of honey as an antimicrobial [6]. Throughout history, honey has been used in a variety of cultures, with differing applications. The ancient Egyptians used honey as a topical ointment, a wound dressing and for embalming their dead, whereas the ancient Greeks used it as a remedy for gout, pain, fever and also wound healing [7]. The first observations of the antimicrobial activity of honey were made in 1892, and since then honey has been observed to have a broad spectrum of activity, inhibiting both Gram positive and Gram negative organisms, including: *Escherichia coli, Pseudomonas aeruginosa, Klebsiella pneuomniae, Staphylococcus aureus, Bacillus subtilis* and *Listeria monocytogens* and their multidrug-resistant counterparts (Table 1) [8,9]. The efficacy of honey against these organisms is dependent on the honey used, due to variations in botanical origin, bee health, geographical location and the processing of honey [1,10,11]. Manuka honey, from the Australian *Leptospermum sp.*, has been identified to inhibit the Gram positive organism *Enterococcus faecalis*, whereas the Gram negative *E. coli* was more resistant to honey treatment [12]. Observations of Manuka and Chinese Buckwheat (*Fagopyrum esculentum*) honey identified a minimum inhibitory concentration (MIC) of 5% (*w/v*) against *S. aureus* and 60% (*w/v*) against *P. aeruginosa* [13]. Similar results of linen vine honey showed *S. aureus* was more susceptible than *P. aeruginosa* [14]. Another study observing the effectiveness of honey from a variety of botanical origins identified greater susceptibility overall towards the Gram positive organisms, *S. aureus* and *Staphylococcus epidermidis*, and either no effect or reduced susceptibility to the Gram negative organisms, *E. coli* and *P. aeruginosa* [15]. Further to this, one study observing the antimicrobial activity of Polish honey against *S. aureus* found an MIC of only 1.56% (*v/v*) of honey was required [16]. However, other studies have identified that Gram positive bacteria are more resistant to honey [17,18,19]. One study identified that Gram negative organisms were more susceptible to honey than Gram positives, suggesting this could be due to the higher hydrogen peroxide content and osmolality of the samples [20]. In regards to Rubus honey, from Southwest Spain, *Proteus mirabilis* was the most susceptible organism tested, exhibiting an MIC range of 7.8 to 31.3 mg/mL, yet *S. aureus* had an MIC range of up to 125 mg/mL [17]. Further to this, honeys of monofloral origin (algarrobo and citrus) and mulitfloral origin exhibited greater efficacy against the Gram negative organisms than the Gram positive organisms, with *P. aeruginosa* having an MIC of 100 mg/mL, whereas *S. aureus* MIC ranged from to 250 mg/mL and *E. feacalis* ranging from 200 to 250 mg/mL with some honey samples having no effect on either Gram positive organism tested [18]. Moreover, a study observing the effect of Egyptian honey identified the only effective honey against *S. aureus* was Sidr honey at an MIC of 100% and only four out of six honey samples were effective against *Streptococcus mutans*. All honey samples tested were effective against *P. mirabilis* and *K. pneumoniae* with MIC values of 50% or less. Only one honey was not effective against *E. coli* and three out of six were not effective against *P. aeruginosa,* but the MIC values for those that were inhibitory were 50% or less [21]. Furthermore, it has been identified that *Acinetobactor calcoaceticus* was the most affected organism, compared to *E. coli, P. aeruginosa* and *S, aureus,* when treated with a range of Scottish honey samples [19]. This variety of results suggests that not all honeys are equal and their effectiveness is largely variable, outlining the significance of botanical origin and geographical location on the antimicrobial activity exhibited by a specific honey. 

**Figure 1:**
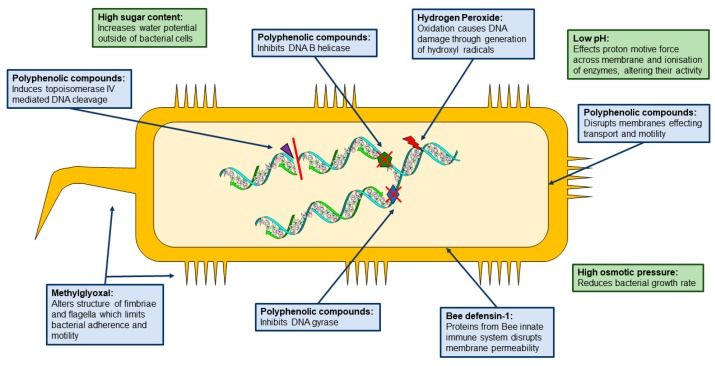
The main constituents attributed to honey’s antimicrobial activity and their mechanism of action. Direct inhibitory factors affect cellular mechanisms (**blue**), indirect inhibitory factors have a wider ranging effect on the bacterial cell (**green**).

Interestingly, it has been observed that no organism has gained resistance to honey [28]. Furthermore, sub inhibitory doses of honey have been shown to restore oxacillin susceptibility in methicillin-resistant *Staphylococcus aureus* (MRSA) [29]. Initial studies into honey have outlined some key factors contributing to its antimicrobial effects, these were high sugar content, low pH, hydrogen peroxide, polyphenolic compounds and the identification of an inhibine (Figure 1) [2,8,30]. Further studies exploring why honey is a powerful antimicrobial identified that inhibine was a 1,2-dicarbonyl compound in the form of methylglyoxal, a potent antimicrobial, found mainly in Manuka honey [31]. More recent studies have also identified a bee-derived protein, bee defensin-1, as a potential antimicrobial component within honey (Figure 1) [25]. This furthers the argument that honey samples contain various antimicrobial compounds and their activity cannot be attributed to a single antimicrobial agent. Moreover, honey contains multiple components that act synergistically, enhancing its potency as an antimicrobial. This review aims to explore the different components that are attributed to honey’s antimicrobial activity and its potential applications. 

## 2. Composition and Classification

Honey is a complex food substance, comprised of 180 to 200 different substances, including sugar, water, proteins, vitamins, minerals, polyphenolic compounds and plant derivatives [10,25]. Depending on origin, honey can be classified as honeydew or blossom. Honeydew honey is produced by the collection of living plant, aphid and insect secretions [32], whereas blossom honey is produced by the collection of flower nectar and characterised by pollen content. Blossom honey can be further divided into unifloral, where the botanical origin is predominantly from one flower species, or multifloral, where multiple sources of flower species can be identified [33]. The botanical origin of honey can have the biggest influence on its antioxidant activity [34]. One honey that has been of great significance, due to its broad spectrum of antimicrobial activity, is Manuka honey, derived from *Leptospermum sp.* [1]. This unifloral honey is used within the pharmaceutical industry and has been developed into medical grade honey. The antimicrobial activity of Manuka honey has been attributed to phytochemicals produced by the *Leptospermum sp.* plant and subsequently transferred to the honey. Recently however, honeydew honey has been investigated as a more potent antimicrobial than unifloral honey, furthering the importance of honey origin [35]. Furthermore, the composition of active compounds present within plant nectar can vary, depending on geographical location and climate conditions [34]. All of these different components can influence the quality of the honey and, subsequently, the antimicrobial activity.

## 3. Carbohydrates

Carbohydrates, predominantly monosaccharides such as glucose and fructose, constitute up to 82.4% of the chemical make-up of all varieties of honey [36]. The next largest component of honey is water, ranging from 13–23% [37]. These two factors impose a stressful environment for microorganisms, as a result of low pH and high osmotic pressure, preventing food spoilage due to unsuitable growth conditions (Figure 1) [2,37]. It is considered that this unfavourable environment largely contributes to the antimicrobial activity of honey. Wahdan (1998) demonstrated that an undiluted sugar solution, mimicking the same sugar and water percentage of honey, exhibited bacteriostatic and bactericidal activity, indicating that these parameters play an important role in the antimicrobial activity of honey [38]. Conversely, Brady, Molan and Harfoot (1996) created an artificial honey, representative of sugar content and acidity, and tested it against a range of dermatophytes, a pathogenic fungus that is the cause of cutaneous mycoses [39]. They observed no inhibitory activity against any organism tested. However, they did observe activity for Manuka honey, suggesting that high sugar levels and low acidity are not the sole source of antimicrobial activity. Further to this, Wahdan (1998) found significant differences between the activity of the sugar solution and honey, indicating there are other components within honey that attribute to its antimicrobial activity [38]. In 1937, the antimicrobial activity of honey was linked to the presence of an ‘inhibine’, a previously unidentified component of honey, the discovery of which supported the theory that sugar content alone was not responsible for the antimicrobial activity exhibited by honey [38]. Studies exploring the mechanisms behind the antimicrobial activity of honey identified a variety of other possibilities, including the presence of polyphenols, hydrogen peroxide 1,2-dicarbonyls and bee defensin-1 (Figure 1). 

## 4. Polyphenolic Compounds:

**Figure 2:**
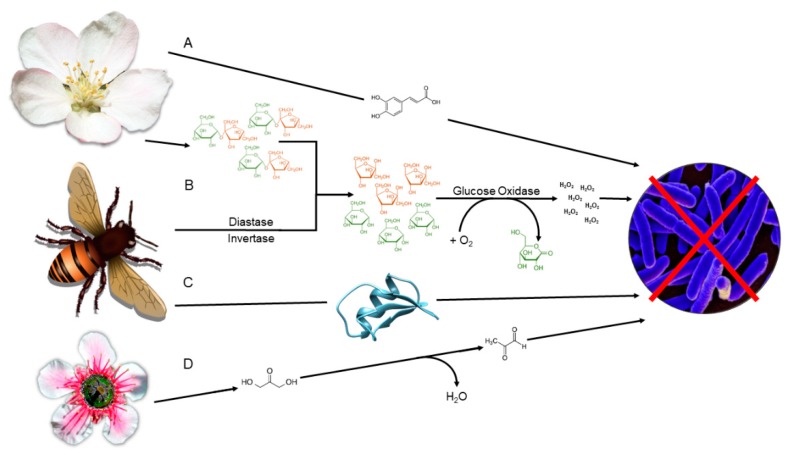
Acquisition of antimicrobial compounds within honey. (**A**) Polyphenolic compounds derived from the plant are transferred by the bee. (**B**) Sucrose from the flower is ingested by the bee and broken down into glucose and fructose upon addition of diastase and invertase by the bee. The glucose is oxidised by glucose oxidase upon the addition of oxygen, producing D-glucono-δ-lactone and hydrogen peroxide. The hydrogen peroxide has antimicrobial activity. (**C**) Bee defensin-1 is added to honey by the bee (Swissmodel 6mry.5.A). (**D**) Dihydroxyacetone is harvested from *Leptospermum sp.* and converted non-enzymatically to methylglyoxal through dehydration reaction.

Polyphenolic compounds are a diverse group of chemicals that include flavonoids and phenolic acids (non-flavonoids), defined by the presence of phenolic structures [40]. Produced as plant secondary metabolites, these bioactive compounds are transferred from the plant to the honey (Figure 2), and have been identified as a major component of the health-promoting properties of honey [41]. Furthermore, the phenolic acids identified in honey have been used to identify the botanical and geographical origin of a given honey sample [17]. Therefore, the botanical origin of honey is significant because it can influence the phytochemicals present, and consequently impact the antimicrobial capacity [11,32]. 

Polyphenolic compounds have been identified in honey, a variety of which have been identified as having antimicrobial activity and the mechanisms of action have largely been elucidated (Table 2, Figure 1). The concentrations at which these polyphenolic compounds are active are much lower within honey, however a similar occurrence has been observed with regards to hydrogen peroxide. Furthermore, polyphenols are typically responsible for destroying free radicals and inhibiting oxidation, and have been suggested to be involved in the generation of hydrogen peroxide [25,42,43,44]. The testing of honey phenolic extracts against *S. aureus, E. coli* and *K. pneumoniae* identified an antimicrobial affect [43]. Further investigations into the role of polyphenolic compounds and their direct antimicrobial impact on honey are required. 

## 5. Hydrogen Peroxide

The presence of hydrogen peroxide within honey has been well established and is considered one of the main antimicrobial constituents in honey. It is produced as a by-product during nectar harvest by the honey bee (*Apis mellifera*). Upon harvest, bee-derived enzymes are added, including diastase, invertase and glucose oxidase. The diastase and invertase break down the larger disaccharides, mainly sucrose, into monosaccharides, glucose and fructose [58]. Upon the addition of oxygen, glucose oxidase catalyses the oxidation of glucose to D-glucono-δ-lactone and hydrogen peroxide, the latter of which has antimicrobial activity (Figure 2) [59]. Interestingly, the antimicrobial effect of hydrogen peroxide in honey increases upon dilution, enabling the glucose oxidase enzyme to bind to glucose more readily, resulting in a continuous production of hydrogen peroxide [24]. It has also been suggested that molecular crowding could play a role in hydrogen peroxide production, provided the concentration of glucose was high enough [60]. The levels of hydrogen peroxide in honey vary between samples and are dependent on two factors: the amount of glucose oxidase added and the presence of pollen-derived catalase [61]. Since glucose oxidase catalyses the reaction, it is assumed that higher levels of glucose oxidase result in more hydrogen peroxide production. This can be influenced by honey bee health and diversity of foraged diet [62]. Conversely, more recent research has suggested that the levels of glucose oxidase present are not directly related to the volume of hydrogen peroxide produced, although these non-enzymatic methods of production are yet to be elucidated [25]. Additionally, catalase is known for the breakdown of hydrogen peroxide into water and oxygen, therefore it is of no surprise that catalase concentration is proportional to hydrogen peroxide content [61]. 

Hydrogen peroxide is a well-established antimicrobial agent. Classed as an oxidative biocide, it removes electrons from chemical structures, resulting in oxidation [63]. The oxidation action causes inhibition of microbial growth and irreversible DNA damage through the generation of hydroxyl radicals [3,64,65]. The generation of hydroxyl radicals in honey is produced in a Fenton-like reaction through hydrogen peroxide. It is noteworthy that, upon the addition of Fe^2+^ or Cu^2+^ ions, an improved bacteriostatic effect against MRSA and VRE (Vancomycin-resistance Enterococci) was observed due to the increased decomposition of hydrogen peroxide to hydroxyl radicals, whereas the removal of hydrogen peroxide with catalase restored bacterial growth, outlining the relationship between hydroxyl radical generation and hydrogen peroxide production [66]. 

Hydrogen peroxide levels within honey can range between 0.5 and 2.5 mM, however, a minimum level of 2.7 mM hydrogen peroxide is required to cause DNA degradation in *E. coli* [60]. Regardless of this, honey containing less than 2.5 mM hydrogen peroxide can exhibit the ability to induce DNA degradation in bacteria, suggesting that hydrogen peroxide is not the only antimicrobial component of honey. A relationship between hydrogen peroxide, polyphenols and DNA degradation induced by honey has been outlined, suggesting higher levels of polyphenols in the presence of hydrogen peroxide improved the oxidative stress imposed on bacterial cells [67]. However, Manuka honey maintains DNA degradation after removal of hydrogen peroxide and exhibits no change in antimicrobial activity, indicating that hydrogen peroxide is not the only antimicrobial component within honey [31,59]. 

## 6. 1,2-dicarbonyls

Antimicrobial activity observed in honey that contains reduced hydrogen peroxide, or after the removal of hydrogen peroxide, has been defined as non-peroxide activity. Non-peroxide activity has been attributed to a variety of different substances, one of which is a group of compounds known as 1,2-dicarbonyls. The 1,2-dicarbonyls are highly reactive compounds, generated in carbohydrate-rich foods through caramelization or Maillard reactions [68]. These are achieved through heat treatment or prolonged storage and are associated with aroma, colour and taste [69]. 1,2-dicarbonyls are formed as an intermediate of a non-enzymatic reaction with glucose and free amino groups, resulting in the formation of advanced glycation end products (AGEs) [70]. Those formed by hexoses include 3-deoxyglucosone (3-DG) and glucoson; formation by disaccharides and oligosaccharides results in 3-deoxypentosone (3-DP) [68]. Breakdown products of 3-DG result in the generation of 5-hydroxymethalfurfural, indicating honey freshness [9]. Other breakdown products of antimicrobial significance are methylglyoxal and glyoxal. 

Methylglyoxal (MGO) has been identified as the main antimicrobial component of Manuka honey [71]. The MGO content of Manuka honey has been directly correlated to the ‘Unique Manuka Factor’ (UMF) rating, indicating this is the main antimicrobial component of Manuka honey [72]. The presence of MGO in Manuka honey is determined by the concentration of dihydroxyacetone. Adams, Manley-Harris and Molan (2009) identified that all nectar collected from *Leptospermum sp.* contains varying levels of dihydroxyacetone and no measurable MGO [73]. To further investigate this, they added dihydroxyacetone to clover honey and observed production of MGO. Furthermore, the addition of arginine and lysine resulted in greater production of MGO, consistent with findings that the non-enzymatic production of MGO requires these amino acids [74]. Within the hive, low amounts of MGO can be detected, but high levels of dihydroxyacetone are present. Once harvested, the conversion of dihydroxyacetone into MGO takes place, resulting in increased MGO levels and a reduction in dihydroxyacetone [75]. Interestingly, heating of the honey to 37 °C results in increased MGO, however, heating to 50 °C causes a loss of both MGO and dihydroxyacetone [73]. 

The conversion of dihydroxyacetone into MGO is considered to happen non-enzymatically in honey (Figure 2). However, in the methylglyoxal pathway, dihydroxyacetone-phosphate is converted to MGO by methylglyoxal synthase [73]. Further research into the production of MGO within honey could elucidate the exact mechanisms behind its production in honey. 

The mechanism of action of MGO is due to its ability to alter the structure of bacterial fimbriae and flagella (Figure 1). Observations were made that increased concentrations of MGO result in less fimbriae and flagella, and a concentration of 2 mM results in the loss of all fimbriae and flagella, as well as inducing damage to cell membranes and the shrinking and rounding of bacterial cells [76]. However, bacteria without fimbriae and flagella have also been observed to be inhibited by Manuka honey, such as *S. aureus*. In Manuka honey, a variety of polyphenolic compounds have been identified, including apigenin, quercetin and caffeic acid (Table 2), which inhibit bacteria through different mechanisms [13]. This further supports that honey possesses multiple antibacterial properties and does not act through a single mechanism. Additionally, these multiple components could be the reason no bacteria have been observed to gain resistance to honey. 

## 7. Bee defensin-1

Bee defensin-1 is an antimicrobial peptide (AMP) identified in bee hemolymph (the bee blood system) and hypopharyngeal glands [6]. It is one of four AMPs, others include apidaecin, abaecin, hymenoptaecin and defensin [77]. Their role within the bee is as an innate immune response, exhibiting activity against fungi, yeast, protozoa and both Gram positive and Gram negative bacteria [78]. Importantly, bee defensin-1 is mainly effective against Gram positive bacteria, most notably *B. subtilis, S. aureus* and *Paenibacillus larvae*, however, it has limited effectiveness against multidrug-resistant organisms [79]. Levels of bee defensin-1 vary between honey samples, this is a result of its production from glands of individual bees, whose production of AMP varies [80]. Although the full mechanism of action for bee defensin-1 has not been elucidated, defensin proteins from other species have been shown to create a pore within the bacterial cell membrane, resulting in cell death [81]. Furthermore, bee defensin-1 has been shown to be important in the role of wound healing, through stimulation of MMP-9 secretions from keratinocytes [82]. 

## 8. Antibiotic Residue

A variety of antibiotic residues have been identified in honey, including sulphonamides, macrolides, tetracyclines and aminoglycosides [83]. The occurrence of this is attributed to the use of antibiotics in apiculture, environmental contamination and improper beekeeping [84]. Within the EU, no trace elements of antibiotics are permitted, however there is no determined maximum residue level and traces can be found in honey samples worldwide [83]. Further to this, it is illegal to use antibiotics in beekeeping in some EU countries. Al-Waili et al., (2012) has suggested that antibiotics in honey could potentially increase the instance of antibiotic resistance, however there is little evidence of this [84]. Increases in antimicrobial resistance are often attributed to the misuse and improper use of antibiotics, as well as their wide applications within the veterinary industry, extending to the meat and dairy industry [85]. One study identified that antibiotic residues in milk were higher than the minimum residue level, and, overall, commercial farms had higher levels of antibiotic residue than local farms [86]. Another study, focused on determining levels of ciprofloxacin, streptomycin, sulphonamide and tetracycline within meat, found levels of ciprofloxacin and streptomycin to be above the MRL (maximum residue level), with the overall traces of all antibiotics ranging between 20.7 and 952.2 µg/kg [87]. However, traces observed within honey are drastically lower than these values and observations have shown these trace amounts diminish over time. One hive treated with lincomycin identified 24 µg of the antibiotic in honey three days after treatment, however traces lowered to 3.5 µg 129 days after treatment [88]. Therefore, the occurrence of antibiotic residue within honey should not be a cause for concern at present. Furthermore, these amounts are sufficiently low that they could not be attributed to the antimicrobial activity observed in honey. 

## 9. Antibiofilm Properties

Biofilms are formed by bacteria upon adhesion to a surface, resulting in the production of an extracellular matrix [89]. This matrix allows for protection of the bacterial community, preventing penetration of antimicrobials and avoiding host defences [90]. Honey has been observed to effectively inhibit and kill a range of planktonic bacteria, but, more interestingly, honey has the ability to disrupt biofilms. The antibiofilm properties of honey have been attributed to its ability to disrupt quorum sensing and penetrate the biofilm itself [91]. Honey has been shown to effectively kill single-species biofilms, including those of *P. aeruginosa, S. aureus* at a one in two dilution, and *Streptococcus pyogenes* at 30% Manuka honey (*w/v*) [92,93]. However, multispecies biofilms are more common, especially in regard to honeys used within a clinical setting, indicating a more important area of antibiofilm research. One study explored the effects of Manuka, honeydew and artificial honey at a concentration of 100% on multispecies biofilms formed of *Streptococcus agalactiae, S. aureus, P aeruginosa* and *E. faecalis*. They identified that Manuka and honeydew honey had antibiofilm efficacy against *P. aeruginosa, S. aureus* and *S. agalactiae*, but no effect was observed from artificial honey against *S. aureus* and *S. agalactiae*. Moreover, all honey varieties were able to successfully inhibit *P. aeruginosa*, including the artificial honey, whereas no sample was able to inhibit *E. faecalis* [94]. This shows promise for the use of honey against multispecies biofilms, especially within wounds. However, the concentration of honey administered needs to be considered, as it has been demonstrated that sub-inhibitory concentrations of honey can improve biofilm formation in *S. aureus*, rather than inhibit it [16,95]. Therefore, more research is required to define the appropriate concentrations of honey to be administered for this purpose.

The ability of honey to disrupt biofilms has been attributed to two main components: bee defensin-1 and MGO [94,96]. The production of biofilms is achieved through external signals, followed by the activation of specific genes [97]. Therefore, the ability of bee defensin-1 to disrupt membranes, resulting in the inhibition of DNA, RNA and protein synthesis, identifies it as an obvious candidate for biofilm disruption [81]. Furthermore, the capability of MGO to alter bacterial fimbriae and flagella, ultimately preventing adhesion to surfaces, would impair biofilm formation [76]. Thus, it is unsurprising that the presence of either bee defensin-1 or MGO results in antibiofilm action. In addition, this suggests that Manuka honey not only has potent antimicrobial activity, but antibiofilm activity as well. 

## 10. Honey and Antibiotic Synergy

Observing the broad spectrum of activity exhibited by honey, especially against drug-resistant organisms, has led to investigations of honey–antibiotic synergy. A variety of antibiotics and honey combinations have now been explored, with some promising results. The pairing of Manuka honey with tetracycline exhibited an increased antimicrobial affect against *P. aeruginosa* and *S. aureus*. The broad spectrum activity of tetracycline, and the enhancement of its activity upon the addition of Manuka honey, makes the combination a strong candidate for wound healing [98]. Another combination, in which sub-inhibitory concentrations of Medihoney were used alongside rifampicin, detected no rifampicin resistance of *S. aureus*, including MRSA and clinical isolates [99]. This is not the first instance of honey reversing resistance to antibiotics. Jenkins and Cooper (2012) identified that sub-inhibitory concentrations of honey, with the addition of oxacillin, restored the susceptibility of MRSA to oxacillin [29]. These findings provide a strong basis for the use of honey in clinical settings, especially for persistent or chronic infections. Additionally, combinations of honey and antibiotics have been shown to have synergistic and additive actions against biofilms. This was demonstrated by the combination of vancomycin with Manuka honey against *S. aureus,* and gentamicin with Manuka honey against *P. aeruginosa* [100]. Furthermore, one study has observed the synergistic effects of Portuguese honey and phage therapy, identifying that 25% (*w/v*) honey paired with phage was equally as effective in *E.* coli biofilm destruction as 50% (*w/v*) honey alone [101]. This highlights the exciting potential and possibilities of the use of honey, and the need for further research into its synergistic effects and clinical applications. 

## 11. Honey in Medical Settings

The main applications of honey within a medical setting are for the treatment of surface wounds and burns. Two distinct types of honey have been developed into medical grade honey, Medihoney and Revamil. Medihoney is developed from Manuka honey, whereas Revamil honey is produced in greenhouses under standardised conditions [80]. Interestingly, the active components of these two honeys differ. The Medihoney activity is based on MGO activity, where hydrogen peroxide activity is variable, with no noted activity of bee defensin-1 [102]. More recently, it has been suggested the defensin-1 in Manuka honey is altered by the presence of MGO, which could have prevented detection of the protein in previous studies [74]. However, Revamil acts primarily through hydrogen peroxide and bee defensin-1 activity [103]. 

The honey can be applied directly to the surface of a wound. This provides a physical barrier between the wound and the environment, preventing contamination [104]. The secondary effects provided by application are the antimicrobial properties, including both bacteriostatic and bactericidal activity, further preventing wound contamination [80]. Additionally, an osmotic gradient is generated due to the high sugar content and low water activity, generating a flow of bacteria, necrotic tissue and debris out of the wound [91]. Finally, the phenolic content in honey aids in inflammation, helping to improve wound healing [105]. Overall, this has been observed to improve both the healing of the wound, and the time taken to heal and reduce scarring [106]. This can reduce the use of antibiotics, while still aiding wound treatment.

A case study involving two patients deployed the use of honey to aid in wound healing and clearing of infection. The first patient had a persistent self-inflicted wound that showed no sign of healing; upon daily treatment with Manuka honey the wound showed signs of re-epithelialisation, and, after six weeks, it had fully healed, demonstrating the ability of honey to promote angiogenesis. The second patient had incurred two large haematomas which became infected with *P. aeruginosa* and *S. aureus*, later confirmed to be MRSA. After failure to heal, Manuka honey was used to clear the infection and promote healing. After eight weeks, the infection had been cleared [107]. Another study which explored the use of honey to aid the healing of skin grafts identified increased healing and reduced pain in comparison to the vaseline control [108]. Additionally, honey can be used to heal burns. In a study observing the effects of honey and 1% silver sulfadiazine, they found that honey reduced the healing time and cleared the burn of infection and pain quicker than the 1% silver sulfadiazine [109]. 

These case studies outline the different uses of honey within a clinical setting, outlining that honey should be implemented in a variety of wound healing applications, not only to prevent infection, but also to reduce healing times and patient discomfort. 

## 12. Conclusions

Honey is a potent antimicrobial agent, exhibiting a broad spectrum of activity. A variety of components contribute to the antimicrobial potential of honey, including sugar content, polyphenolic compounds, hydrogen peroxide, 1,2-dicarbonyl compounds and bee defensin-1. All of these are present in varying levels, depending on nectar source, honey bee and storage. These components act synergistically, allowing honey to be effective against a variety of microorganisms. The variation in the quantity and structural modifications of components is also a major contributing factor as to why some honeys can be more effective at inhibiting bacterial growth than others, furthering the requirement for continued research. 

Within a medical setting, honey can be used as an effective wound treatment, removing the need for antibiotics. Honey has the potential to vastly reduce the requirement of drugs of last resort for highly drug-resistant bacterial infections, since current resistance to antimicrobial mechanisms of honey is largely unseen. This is likely due to the multiple mechanisms of antibacterial action from the plethora of antimicrobial compounds, resulting in a unique combination therapy, which has yet to be identified as a source of antimicrobial resistance. As the authors of this review, we feel that the use of honey will likely be expanded on in the future. This is largely due to the rise in MDR organisms causing infections that are extensively untreatable by multiple classes of antibiotic, particularly since honey has been shown to be capable of reversing certain mechanisms of antibiotic resistance. Therefore, the revival of this alternative antimicrobial agent represents a promising therapeutic avenue to help curb the increasing incidence of antibiotic-resistant bacterial infections. Furthermore, the complete elucidation of the mechanisms of activity and synthesis of all components of honey could lead to the generation of an optimally antimicrobial synthetic or semisynthetic honey. Discovery of the precise concentrations of these synergistic components would enable us to develop the most effective, broad-spectrum honey with activity against a wide range of antibiotic-resistant bacterial species.

## Figures and Tables

**Table 1:** Antimicrobial effect of honey from different geographical locations.

Geographical Variation in Honeys Antimicrobial Activity		
Country of Origin	Honey Sample	Organisms
**Australia**		
New Zealand [13]	Manuka	*Staphylococcus aureus, Pseudomonas aeruginosa*
New Zealand [22]	Manuka	*S. aureus*, MRSA, MSSA, coagulase-negative *Staphylococcus epidermidis*, *Klebsiella pneumonia*, ESBL *E. coli*
Australia [23]	*Leptospermum* based honey	*S. aureus*
**North America**		
Canada [24]	Canadian Honey	*E. coli, Bacillus subtilis*
Cuba [14]	Christmas vine, Morning glory, Black mangrove, Linen vine, Singing bean	*S. aureus, P. aeruginosa, E. coli* and *B. subtilis*
**South America**		
Chile [11]	Ulmo Honey	MRSA, *E. coli* and *P. aeruginosa*
Argentina [18]	Algarrobo, citrus and multifloral honey	*S. aureus, Enterococcus faecalis, E. coli, Morganella morganii* and *P. aeruginosa*
**Europe**		
Scotland [19]	Blossom, heather, Highland, Portobello Orchard	*Acinetobactor calcoaceticus, S. aureus, P. aeruginosa* and *E. coli*
Northwest Spain [17]	Rubus Honey	*S. aureus, S. epidermidis, Micrococcus luteus, E. faecalis, B. cereus, Proteus mirabilis, E. coli, P. aeruginosa* and *Salmonella. typhimurium*
Denmark [15]	Heather, Rasberry, Rapeseed, Hawthorn and White Clover	*S. aureus, P. aeruginosa* and *E. coli*
Slovakia [25]	Honeydew Honey	*P. aeruginosa* and *S. aureus*
**Asia**		
China [13]	Buckwheat Honey	*S. aureus* and *P. aeruginosa*
Saudi Arabia [26]	Sider Honey	*S. aureus, Streptococcus pyogenes, Corynebacteria pseudotuberculosis, K. pneumonia, P. aeruginosa* and *E. coli*
**Africa**		
Algeria [9]	Astragalus, Wall-rocket, Eucalyptus, Legume, Peach, Juniper, Buckthorn and multifloral	*Clostridium perfringens, S. aureus, E. coli* and *B. subtilis.*
Nigeria [27]	Wildflower and Bitter leaf Honey	*S. typhimurium, Shigella dysenteriae, E. coli, B. cereus* and *S. aureus*
Egypt [21]	Cotton, Blackseed, Orange, Eucalyptus, Sidr and Clover Honey	*E. coli, S. aureus, Streptococcus mutans, P. mirabilis, P. aeruginosa* and *K. pnuemoniae*
Egypt [26]	Acacia, Citrus, Clover, Coriander, Cotton and Palm Honey	*S. aureus, S. pyogenes, Corynebacteria pseudotuberculosis, K. pneumonia, P. aeruginosa* and *E. coli*

**Table 2:** Common polyphenolic compounds found within honey and their antimicrobial mechanism of action.

Phenolic Acids	Mechanism	Flavonoids	Mechanism
**2-*cis,A-trans* Abscisic acid**	Unknown	**Apigenin**	Inhibits DNA gyrase [44]
**2-Hydroxycinnamic acid**	Unknown	**Catechin**	Hydrogen peroxide generation [45]
**Caffeic acid**	Oxidative Stress [46]	**Chrysin**	Inhibits DNA gyrase [47]
**Chlorogenic acid**	Increase in membrane permeability resulting in cytoplasmic and nucleotide leakage [48]	**Galangin**	Inhibition of peptidoglycan and ribosome synthesis [49]
**Cinnamic acid**	Unknown	**Genistein**	Disruption to topoisomerase-II DNA cleavage complex [50]
**Ellagic acid**	Unknown	**Isorhamnetin**	Unknown
**Ferulic acid**	Cell membrane dysfunction and changes in cell morphology [51]	**Kaempferol**	Inhibits DNA gyrase [47]
**Gallic acid**	Cell membrane disruption resulting in pore formation and intracellular leakage [52]	**Luteolin**	Inhibits FAS-I in Mycobacteria and inhibits DNA helicase DnaB and RecBCD [47]
***p*-Coumaric acid**	Cell membrane disruption and binding to bacterial DNA [53]	**Myricetin**	Inhibits DNA B helicase [54]
***p*-Hydroxybenzoic acid**	Unknown	**Naringenin**	Unknown
**Protocatechuic acid**	Unknown	**Pinobanksin**	Unknown
**Sinapic acid**	Unknown	**Pinocembrin**	Induces cell lysis [47]
**Syringic acid**	Cell membrane dysfunction [55]	**Quercetin**	Disrupts membranes, transport and motility [56]
**Vannilic acid**	Unknown	**Rutin**	Induces topoisomerase IV mediated DNA cleavage [57]
